# Optimization and Efficiency Enhancement of Modified Polymer Solar Cells

**DOI:** 10.3390/polym15183674

**Published:** 2023-09-06

**Authors:** Muhammad Raheel Khan, Bożena Jarząbek

**Affiliations:** Centre of Polymer and Carbon Materials, Polish Academy of Sciences, Sklodowska-Curie 34 Str., 41-819 Zabrze, Poland

**Keywords:** polymer thin films, BHJ organic solar cells, hole transport layer (HTL), electron transport layer (ETL), reflective coating, SCAPS-1D simulation

## Abstract

In this study, an organic bulk heterojunction (BHJ) solar cell with a spiro OMeTAD as a hole transport layer (HTL) and a PDINO as an electron transport layer (ETL) was simulated through the one-dimensional solar capacitance simulator (SCAPS-1D) software to examine the performance of this type of organic polymer thin-film solar cell. As an active layer, a blend of polymer donor PBDB-T and non-fullerene acceptor ITIC-OE was used. Numerical simulation was performed by varying the thickness of the HTL and the active layer. Firstly, the HTL layer thickness was optimized to 50 nm; after that, the active-layer thickness was varied up to 80 nm. The results of these simulations demonstrated that the HTL thickness has rather little impact on efficiency while the active-layer thickness improves efficiency significantly. The temperature effect on the performance of the solar cells was considered by simulations performed for temperatures from 300 to 400 K; the efficiency of the solar cell decreased with increasing temperature. Generally, polymer films are usually full of traps and defects; the density of the defect (N_t_) value was also introduced to the simulation, and it was confirmed that with the increase in defect density (N_t_), the efficiency of the solar cell decreases. After thickness, temperature and defect density optimization, a reflective coating was also applied to the cell. It turned out that by introducing the reflective coating to the back side of the solar cell, the efficiency increased by 2.5%. Additionally, the positive effects of HTL and ETL doping on the efficiency of this type of solar cells were demonstrated.

## 1. Introduction

Organic semiconductors and polymers have gained attention for many years as interesting materials suitable for optoelectronics, especially for organic solar cells. Among the renewable energy technologies, the third-generation solar cell is the finest technology and has the potential to replace silicon-based solar cells [1,2,3,4,5]. Organic semiconductors have advantages comparable to silicon solar cells, i.e., light weight, flexible, low cost, and tunable processing at room temperature [6,7]. The silicon solar cells efficiency and stability are still greater than those of organic solar cells (OSCs), but, in the future, these may be increased by using a suitable absorber layer. To improve the power conversion efficiency (PCE) of OSCs, different materials and methods have been used by researchers. The efficiency enhancement methods include: (a) controlling the morphology and surface interactions in the active layer [8,9], (b) development of low-band-gap organic materials to improve the absorption of light in the visible and near-infrared regions [10].

In OSCs, the HTL and ETL are essential components along with the active layer. The HTL facilitates the movement of the positive charge carriers (holes), generated by the absorption of light in the active layer, towards the anode. These layers usually consist of materials that can efficiently transport holes and have good compatibility with the adjacent layers. The ETL, on the other hand, enables the movement of the negative charge carriers (electrons), generated in the active layer, towards the cathode. Like the HTL, the ETL is designed with materials that have excellent electron mobility and interface compatibility. The active layer consists of photoactive materials. When light is incident on this layer, it absorbs photons and generates electron–hole pairs. The efficient separation and transportation of these charge carriers to their respective electrodes (anode and cathode) by the HTL and ETL lead to the conversion of light energy into electrical energy in the OSCs. Some scientists have reached the conclusion that by using an appropriate combination of ETL, HTL, and a blend of donor and acceptor materials as an active layer, an excellent photovoltaic response may be achieved [11,12,13]. The scientific community has shown significant interest in BHJ solar cells due to their numerous advantages, such as being lightweight, cost-effective, having a tunable band gap, and displaying relatively good power conversion efficiency (PCE) [14]. The non-fullerene acceptor (NFA) has also gained considerable attention from researchers, thanks to it achieving higher than 19% efficiency [15,16]. Compared to the fullerene acceptor (FA), the NFA overcomes all the drawbacks observed in the FA when integrated into a BHJ structure. The NFA offers an easily synthesized process, exhibits good visible and near-infrared (NIR) absorption, and according to Ma et al. [17], it boosts long-term stability, unlike the FA.

A group of researchers [18] has developed an OSC incorporating an NFA by employing PBDB-T as a donor while ITIC-OE is used as an acceptor. The efficiency of this solar cell is reported as 8.5% [18]. By adding oligoethylene (OE) side chains to the blend, the NFA dielectric constant can be improved to 6.1. Generally, organic semiconductors have high exciton-binding energy, due to the low dielectric constant values which increase the recombination at the interface [18,19].

Different photovoltaic organic BHJ structures have been investigated and presented in numerous publications. Shabaz et al. presented work [20] on a new energetic indandione-containing planar donor to enhance the stability and efficiency of OSCs. In this article, they presented a novel and energetic indandione-containing donor molecule to boost the performance of the BHJ. The H3T-1D donor molecule had an optical band gap (E_g_) of approximately 1.98 eV, while [6,6]-phenyl C61 butyric acid methyl ester (PC61BM) was utilized as acceptor. The photovoltaic characteristics of the BHJ-based solar cells were measured and discussed in [20], where the fabricated solar cell had a PCE of 4.05%, short current density (Jsc) of 10.43 mA/cm^2^, open circuit voltage (Voc) of 0.77V, and 0.51% fill factor (FF). Another paper [21], concerning a symmetric benzo-selena-diazole-based donor–acceptor–donor (D-A-D) molecule for BHJ-solution processed OSCs, was presented by the researchers. In this work, they synthesized a novel organic compound with a D–A–D structure (RTh-BSe-ThR). Benzoselenadiazole was used as an acceptor while hexylbithiophene was employed as a donor unit by multistep synthetic pathways. The synthesized RTh-BSe-ThR chromophore exhibited improved absorption with a relatively lower E_g_ of approximately 1.87 eV and had optimum HOMO/LUMO energy levels. The experimental results showed that the addition of Se atoms to the chromophore improved the relevant photovoltaic performance parameters of the BHJ OSCs. The RTh-BSe-ThR chromophore was blended with PC61BM in different ratios, such as 1:1, 1:2, 1:3 and 1:4 *w*/*w* and the photovoltaic parameters for the different ratios were calculated. The optimized and highest efficiency was obtained by using 1:3 *w*/*w* with Jsc 11.2 (mA/cm^2^), Voc 6.684, FF 45% and PCE 3.61%. The highest efficiency was due to the light harvesting ability of the active layer, exciton dissociation and charge transport active layer interface. Spiro-OMeTAD is mostly used as a small molecule in organic photovoltaic devices (OPV). Firstly, spiro OMeTAD was used as a solid electrolyte in dye-sensitized solar cells [22] and later in perovskite solar cells (PSCs) [23]. The spiro OMeTAD has the following characteristics such as morphological good stability, and high glass transition temperature (Tg). The highest efficiency, i.e., 25%, was recorded by using spiro OMeTAD as an HTL for PSCs [24].

Compared to experimental work, simulation studies are also important. Through simulation study, we investigate how the device parameters can affect the physical properties and how the solar cells performance can be improved. Much of the available literature is now related to simulation studies for third-generation solar cells; however, some rare research studies have been conducted on OSC simulation. Abdelaziz et al. [25] presented a research paper on the potential efficiency enhancement of an NFA-based solar cell through device simulation. NFAs are attracting attention from the scientific community owing to their relatively high stability and efficiency. SCAPS simulation was used in [25] to perform an analysis of the J-V characteristics and these values were compared to the available literature. The simulation of solar cells showed higher efficiency, i.e., 14.25%, which is close to the reported literature values. The matching characteristics confirm that SCAPS software can be used as a standardized tool for simulation studies of OSCs, including NFA. In [1], the authors developed and optimized a novel design of poly [[4,8-bis [(2-ethylhexyl)oxy]benzo [1,2-b:4,5-b’]dithiophene-2,6-diyl][3-fluoro-2-[(2-ethylhexyl)carbonyl]thieno [3,4-b]thiophenediyl]] [6,6]-phenyl-C71-butyric acid methyl ester (PTB7: PC70BM) by using PEDOT:PSS as an HTL while the poly[(9,9-bis(3′-((N,N-dimethyl)-N-ethylammonium)-propyl)-2,7-fluorene)-alt-2,7-(9,9-dioctylfluorene)] dibromide (PFN: Br) was used as an ETL. They concluded that the optimized solar cell had a Jsc 16.434 mA/cm^2^, FF 68.055%, Voc 0.731 and the efficiency was reported as 8% [1]. Polymers are full of traps, so in this simulation, they introduced defect density in the cell layers to yield more realistic simulations. Graphene oxide (GO) has gained attention due to its remarkable electrical, optical, and mechanical characteristics in solar-cell research [26,27,28]. In [26], the researchers used two absorber layers in their study, PBDB-T/ITIC and PTB7:PC70BM, to find which interacted more strongly with GO. Numerical simulation was performed using SCAPS software by varying the thickness of the absorption layer, defect density, and doping values and the solar cell was optimized to achieve the best possible efficiency for a PBDB-T/ITIC solar cell by using GO; the efficiency was reported as 17.34% [26]. These simulation results provide a path for GO-based solar cell development. Saqib et al. [27] treated a GO/PEDOT:PSS bilayer with UVO (ultraviolet ozone) for various durations of time, i.e., 0, 5, 10 and 15 min. It was observed that the OSC treated for 10 min showed better performance and efficiency was enhanced, i.e., 5.24%. The enhanced efficiency is due to the improvement in Voc 10.82 mA/cm^2^ and FF 57%. In [28], the authors used graphene oxide as HTL. The authors concluded that by using GO as the HTL, while carbon was used as a back contact, an efficiency of 16.5% could be achieved. In [29], the authors performed a simulation study on inverted organic solar cells. It was observed that the PCE increased from 4.88 to 5.7% by applying P3HT and PTB7, and further increased by replacing PEDOT:PSS with MoO_3_, up to 5.92% in the best case. They suggested that increasing the efficiency above 10% could be obtained by nanocomposite or nanoparticle additions to the active polymer layer. Mostly, poly(3,4-ethylenedioxythiophene) polystyrene sulfonate (PEDOT:PSS) is commonly used as an HTL in conventional solar cells because of its better conductivity and improved transparency. However, PEDOT:PSS is acidic in nature and degrades the device. Nithya et al. [30] replaced the PEDOT:PSS with copper iodide (CuI) and carried out a simulation study for PBDB-T: ITIC by employing SCAPS–1D software. The efficiency for this optimized structure was reported as 15.68% and these values are encouraging, and probably CuI can be used soon in real structures as HTL.

The aim of this paper is to investigate the performance of thin film (PBDB-T: ITIC-OE)-based OSCs. ITIC-OE shows similar optical properties to ITIC while the dielectric constant of this version with oligoethylene (OE) is higher than for ITIC. A higher dielectric constant reduces the recombination rate of charge carriers and increases the overall performance in organic solar cells, which results in the increase in the photovoltaic properties, namely Voc, Jsc and PCE. In previous studies, the (PEDOT:PSS) layer in photovoltaic structures was widely used, while in our study, we replaced PEDOT:PSS with the spiro OMeTAD-type HTL. PEDOT:PSS is acidic in nature and has stability problems in the PV structure. In this work, the solar cells active-layer thickness, defect density, HTL and ETL doping levels are optimized to achieve better efficiency. Temperature has a noticeable effect on the performance of solar cells, so simulations were carried out for different temperatures from 300 to 400 K. To the best of our knowledge so far, no studies have been found regarding efficiency improvement through reflective coating for high dielectric constant OSCs. In this work, a back-side reflective coating was introduced, which increased efficiency by 2.5%.

## 2. Materials and Methods

### 2.1. SCAPS-1D Software and Mathematical Modeling

Several types of simulation software can simulate the design of photovoltaic systems, i.e., SAM, RET-Screen and PV-syst. For third-generation solar cells, the most popular and useful is SCAPS-1D software. This special software was invented by Ghent University Belgium to simulate the J-V characteristics and quantum efficiencies [31]. SCAPS-1D is free and reliable open access software. Many authors have presented their simulation as well as experimental results for polymer or organic materials by using polymer as an absorber or ETL/HTL layer. Therefore, we chose the SCAPS-1D software for the simulation of polymer thin film BHJ solar cell. To evaluate the output performance of the solar cells, the following mathematical equations are used [32,33].

Poisson Equation:

(1) $$\frac{\partial^{2}\varphi }{\partial^{2}x}=-\frac{\partial E}{\partial x}=-\frac{\rho }{\varepsilon_{s}}=-\frac{q}{\varepsilon_{s}}[p-n+N_{D}\left(x\right)-N_{A}\left(x\right)\pm N_{def}\left(x\right)]$$

Transport Equation

(2) $$J_{n,p}=nq\mu_{n}E+qD_{n}\frac{\partial n}{\partial x}+pq\mu_{p}E+qD_{p}\frac{\partial p}{\partial x}$$

Continuity Equation

(3) $$\frac{\partial n,p}{\partial t}=\frac{1J_{n}}{q\partial x}+\left(G_{n}-R_{n}\right)+\frac{1J_{p}}{q\partial x}+\left(G_{p}-R_{p}\right)$$

Diffusion Length

(4) $$L_{n,p}=\sqrt{D_{p,n}\tau_{n,p}}$$

Diffusivity

(5) $$D_{p,n}=\left[\left(\frac{K_{B}T}{q}\right)\mu_{n,p}\right]$$

Open circuit voltage

(6) $$V_{OC}=\frac{{nK}_{B}T}{q}\left[I_{n}\left(\frac{I_{L}}{I_{O}}+1\right)\right]$$

In the equations, φ and ε represent electrostatic potential and permittivity, respectively, while *ρ* and q represent density and elementary charge, E is an electric field. N_D,_ N_A_ represent densities of donor and acceptor.

Symbols D_n_, D_p_, J_n,p_, μ_n_, μ_p_ represent electron and hole diffusion coefficients, current densities, and motilities, G_n_ and G_p_ represent the generation rate for electron and hole, while recombination rate for electron/hole is represented by R_n_ and R_P,_ respectively. The electron/hole lifetime is described by τ_n,p_ and the expression $\frac{K_{B}T}{q}$ represents the thermal voltage, I_L_ and I_O_ represent current generation by light and saturation current, respectively.

### 2.2. Device Architecture

**Figure 1:**
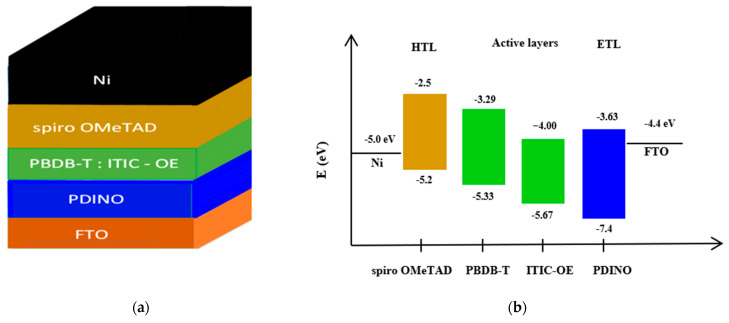
(**a**) General structure for simulation along with HTL, ETL, active layers; (**b**) energy states of OSC layers.

The herein investigated organic thin-film solar cells have five layers which are shown in Figure 1. The first layer is the cathode; its function is to collect electrons. Then comes ETL; its basic function is to collect electrons from the absorber. The ETL is also known as the hole block layer. The absorber is the third layer (known as an active layer); when the light falls on the absorber, charge carriers are produced; charge carriers are the combination of electron and hole. The fourth layer is the HTL which is also used as the electron blocking layer. It serves to extract the holes from the active layer. The final layer serves as an anode, with the function of collecting holes.

Poly(2,2′-disulfonyl-4,4′-benzidineterephthalamide: (3,9-bis(2-methylene-(3-(1,1 dicyanomethylene)-indanone)-5,5,11,11-tetraki (4-hexylphenyl)-dithieno [2,3-d:2,3-d]-s-indaceno [1,2-b:5,6-b]dithiophene oligoethylene)) (PBDB-T:ITIC-OE) was used as an active layer (active layer is the blend of donor and acceptor materials). PBDB-T was used as a donor material while ITIC:OE (oligoethylene side chain) was used as an acceptor. 2,2′,7,7′-Tetrakis[N,N-di(4-methoxyphenyl)amino]-9,9′-spirobifluorene (spiro OMeTAD) was applied as an HTL while N,N’-Bis(N,N-dimethylpropan-1-amine oxide)perylene-3,4,9,10-tetracarboxylic diimide (PDINO) was used as an ETL. Ni film served as an anode while FTO was used as a cathode. The general structure and energy state diagram of the proposed organic solar cell is shown in Figure 1.

### 2.3. Parameters Used in the Simulation

All the values which were required for the simulation were collected from the literature. These values included band gap, electron and hole mobility, doping density, electron affinity, defect density for HTL, ETL and for the active layer blend. All the simulations were performed under the standard test conditions (STCs). The standard conditions are air mass of 1.5 G, temperature 300 K and irradiance 1000 W/m^2^. The voltage value was set up to 1.3 V. The simulation parameters for active layer, HTL and ETL are presented in Table 1 while parameters for back contact and front contact electrodes are presented in Table 2 and Table 3, respectively.

## 3. Results and Discussions

### 3.1. Current Density-Voltage (J-V) Characteristics and the Energy-Band Alignment

**Figure 2:**
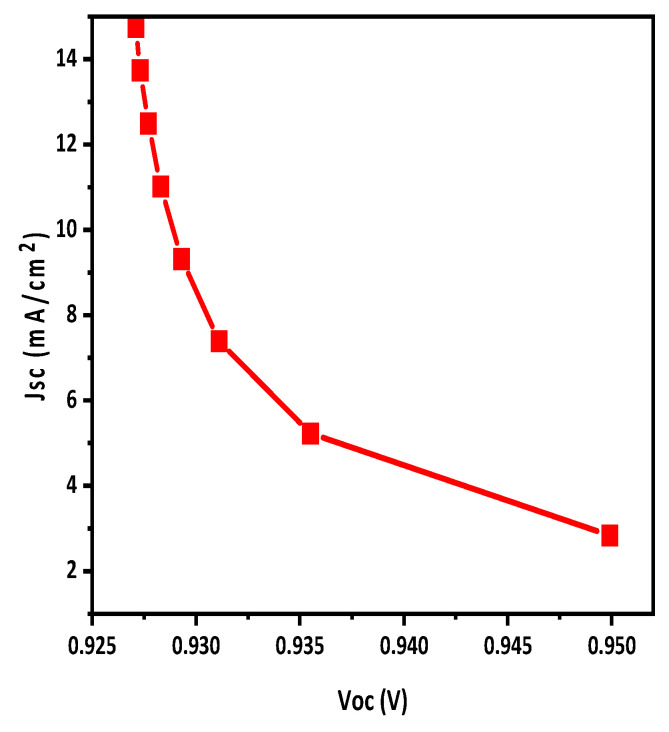
J-V characteristics curve of simulated structure; short circuit current density (*J*_SC_) is shown as a function of open circuit voltage (*V*_OC_).

The J-V characteristics curve for the proposed structure was obtained and is shown in Figure 2, while the output performance parameters are shown in Table 4. Firstly, the results were obtained for the optimized structure and then the reflective coating was applied to enhance the efficiency of this solar cell.

**Figure 3:**
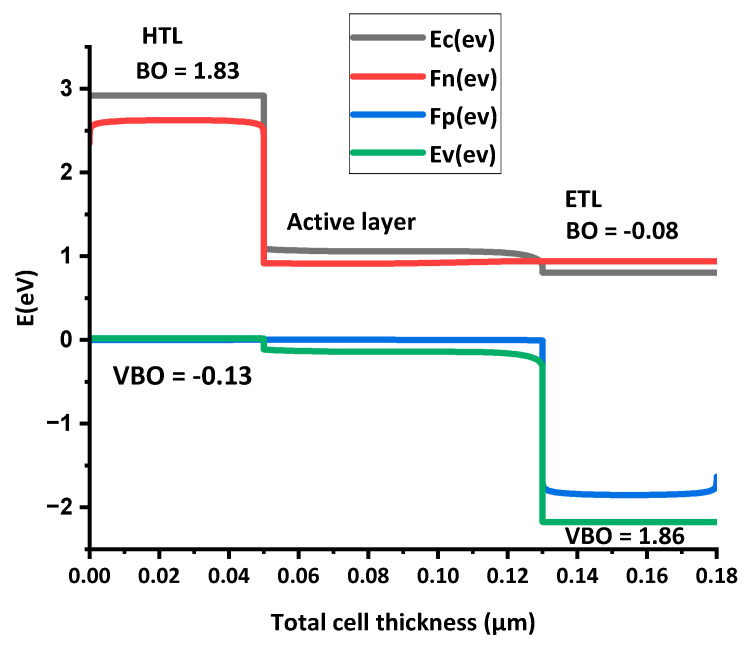
Energy-band alignment of the HTL and ETL, along with the active layer.

Energy-band alignment is critical to the performance of solar cells. Figure 3 contains the band-alignment diagram for the HTL and ETL along with active layer. It shows that the HTL has maximum conduction-band offset (CBO) and minimum valence-band offset (VBO). On the other hand, the ETL has minimum CBO and maximum VBO.

Table 5 presents the CBO and VBO values of HTL and ETL. In Figure 3, E_c_ represents conduction-band offset, F_n_, Fermi level of electrons, F_p_, Fermi level of holes and E_v_, valance-band offset.

- **E_c_**—in a semiconductor, the conduction band comprises the lowest occupied molecular orbital (LUMO). It is the highest energy level where the electrons can move freely within the solid [37].
- **F_n_**—represents the Fermi level of the n-type material, where the probability of an electron is 0.5. The Fermi level acts as a boundary; it distinguishes the energy levels where there is a high likelihood of the existence of electrons from those where their presence is relatively unlikely. In an ETL or in n-type semiconductor, the F_n_ is close to the conduction band due to a surplus of electrons [37].
- **F_p_**—represents the Fermi level of the holes, which is located near the valence band due to surplus of holes [37].
- **E_v_**—in a solid, the valence band represents the lowest energy level, where the electrons are tightly bound to the atoms and lack freedom of movement. In solar cells, this VB is composed of the semiconductor material’s highest occupied molecular orbital (HOMO) [37].

When the CB of the charge transport layer (CTL) and active layer are aligned, then there will be no hurdles in the way of the electron and the flow will be smooth. If the active layer CB is above the CB of ETL, it forms a cliff interface which means negative conduction-band offset (−CBO). The negative CBO reduces the performance of the solar cell. If the conduction band of the active layer is below the conduction band of the HTL, then a spike interface is formed. Spike interfaces mean the (+CBO) electron will flow smoothly but spikes can cause hurdles for the electron flow, and recombination will occur.

Similarly, for the smooth flow of holes, the CTL valence band (VB) and active layer VB are aligned; if the active layer VB is below the VB of the HTL, then a cliff interface is formed (−VBO). The negative VBO reduces the performance of OSCs. If the active layer VB is above the VB of the ETL, then the spike interface is formed. These spike interfaces improve the performance of the solar cell. The CBO and VBO of the HTL and ETL along with active layer can be calculated by the Anderson rule. According to the Anderson rule, the offset can be calculated as [32]:

(7) $$CBO=(\chi_{activelayer}-\chi_{CTL})$$

In the above formula, χ represents electron affinity.

(8) $$VBO=(\chi_{CTL}-\chi_{activelayer}+{Eg}_{CTL}-{Eg}_{activelayer})$$

In the above formula, E_g_ represents the energy gap of the CTL and the active layer, respectively.

The ideal band gap alignment of the solar cell has maximum CBO of HTL and minimum CBO of the ETL, but minimum VBO of the HTL and maximum VBO of the ETL.

### 3.2. Impact of Active-Layer Thickness on OSC

**Figure 4:**
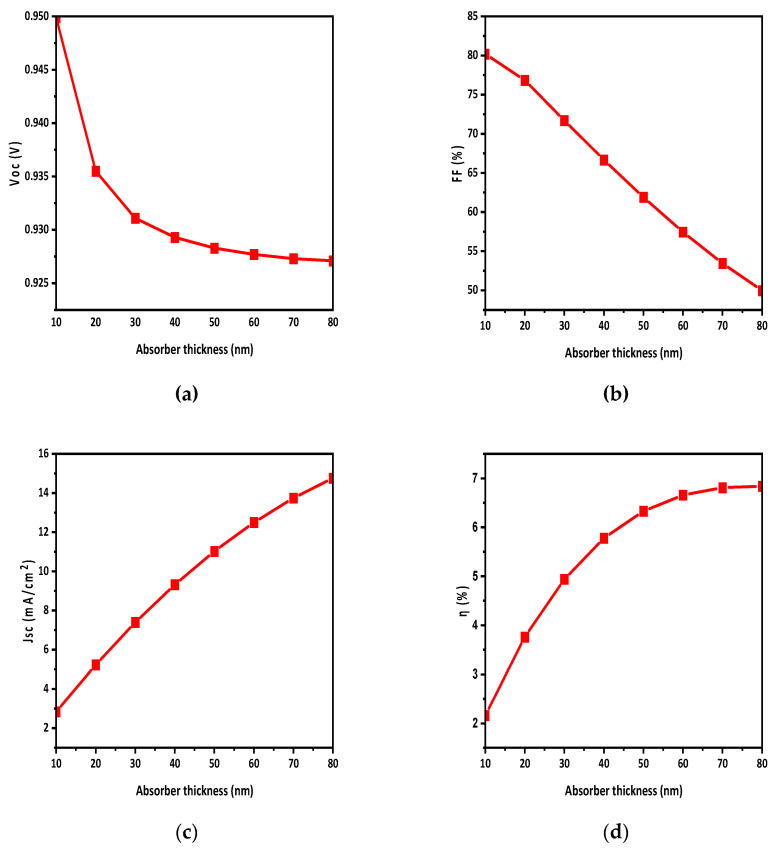
Effect of active-layer thickness on photovoltaic parameters: (**a**) Voc; (**b**) FF; (**c**) Jsc; (**d**) η.

The thickness of the HTL was optimized up to 50 nm at the start; however, it turned out that the HTL thickness had little influence on the device performance while the ETL layer thickness was kept constant, i.e., 50 nm. The thickness of the absorber is a critical factor in the photo absorption and charge generation process. When the film thickness is insufficient, it exhibits characteristics like a transparent film. Hence, we altered the thickness of the active layer in our simulation study, with a range from 10 to 80 nm. The main purpose of this investigation was to assess its influence on solar cell performance and to enhance the electron-hole creation process. Figure 4 illustrates how the parameters change with varying active-layer thickness. Specifically, it was observed that Voc and FF decrease while η and Jsc increase, as depicted in Figure 4a,b and Figure 4c,d, respectively. The decrease in Voc with increasing active-layer thickness can be attributed to several factors. Firstly, thicker active-layer charge carriers have to travel a longer distance from the photoactive layer to the respective electrodes, leading to increased resistance. Additionally, thicker layers may contain traps and defects that further reduce Voc. The decrease in FF is primarily due to an increase in series resistance, as the active-layer thickness grows. This increase in resistance losses within the device can be attributed to factors such as the longer distance for charge carriers to travel, charge-carrier trapping, and an increase in contact resistance at the electrodes. Despite the decrease in FF and Voc, the overall increase in the η and Jsc of the solar cell is due to the increase in light absorption and the generation of charge carriers.

### 3.3. Active-Layer Defect Density Effect on The Performance of Solar Cells

The performance and outcomes of OSCs are significantly influenced by the structure and quality of the active layer. The defect density in the device is a crucial factor in achieving efficient results. Since polymers typically contain traps, our simulation incorporated a realistic N_t_ to accurately represent the system. The N_t_ depends on two parameters: (1) the quality of the active layer and (2) the preparation technique employed. A higher N_t_ value indicates an increased presence of light traps and crystal defects within the active layer. These traps hinder the movement of charges and promote recombination, ultimately affecting the solar cells performance. Additionally, a higher N_t_ also influences the lifetime of the charge carriers, causing a reduction in their longevity. Mathematically, this relationship is expressed as [33]:

(9) $$\tau =\frac{1}{\sigma *N_{t}*V_{\mathrm{th}}}$$

In Equation (9), σ represents the capture cross-section, N_t_ stands for defect density and V_th_ denotes the thermal velocity associated with the carriers. The defect density (N_t_) plays a crucial role in determining the recombination rate, known as the Shockley–Read–Hall recombination (R), which can be expressed as [38,39,40]:

(10) $$R=\frac{np-{ni}^{2}}{\tau p\left(n+nt\right)+\tau n(P+Pt)}$$

where ni denotes intrinsic carrier concentration, n and np represent mobile-carrier concentration and Pt refers to the trap-defect concentration.

**Figure 5:**
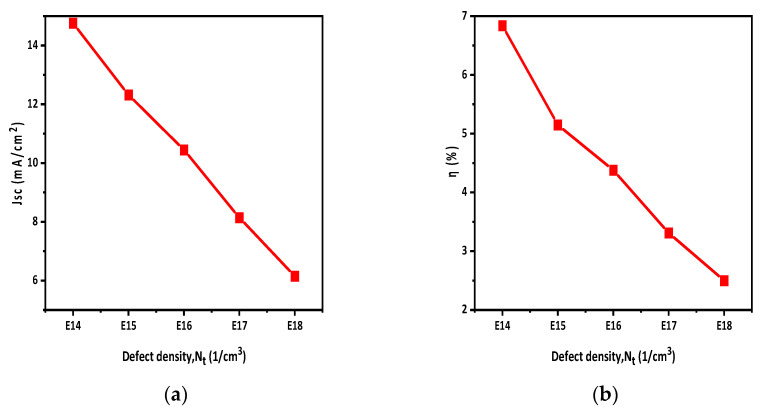
The graphs show how the active-layer defect density influences photovoltaic parameters (**a**) Jsc; (**b**) η.

To investigate the impact of defect density (N_t_) on the OSC structure, we varied N_t_ values from E14 to E18. Figure 5a,b illustrates the influence of defect density on the Jsc and overall solar cell η. As the defect density (N_t_) increases, there is a noticeable decrease in solar cell η. After analyzing the results, we found that the optimized N_t_ value for this structure was E14.

### 3.4. Effect of Temperature

**Figure 6:**
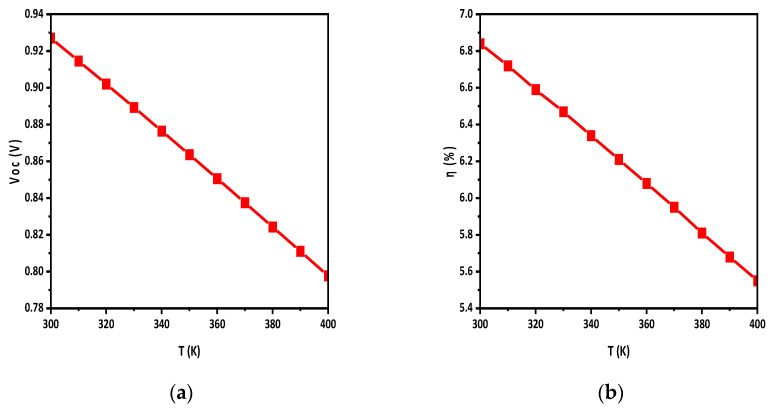
Effect of temperature on (**a**) Voc; (**b**) η.

Solar cells are mostly exposed to higher temperatures than ambient, thus understanding the temperature impact on performance is essential. The ideal temperature for operating solar cells is room temperature; however, the real temperature may vary between 300 and 400 K or maybe higher, so this simulation was performed by stepping up the temperature from 300 to 400 K. It was determined from the simulation results that temperature variations have an influence on the OSCs’ performance. Figure 6a,b illustrates how temperature affects V_oc_ and η.

The decrease in Voc is due to the reverse saturation current density. Additionally, this phenomenon is further linked to the intrinsic carrier concentration (*ni*) and *ni* is related with band gap by the following mathematical relationship [41].

(11) $${ni}^{2}={K1e}^{-E_{g}/KT}$$

where *K*1 is a constant.

*E_g_* varies inversely proportionally to the temperature as expressed by the mathematical equation [41].

(12) $$E_{g}\;\left(T\right)=E_{g}\;\left(0\right)-\frac{\alpha T^{2}}{T+\beta }$$

*α* and *β* indicate the material constants, while *E*_g_(0) refers to the band gap at temperature zero Kelvin.

Generally, a higher temperature means that more electrons will be excited, so the band gap may be unstable, and the recombination of carriers increases and, as a result, the efficiency decreases.

### 3.5. Effect of Reflective Coating

**Figure 7:**
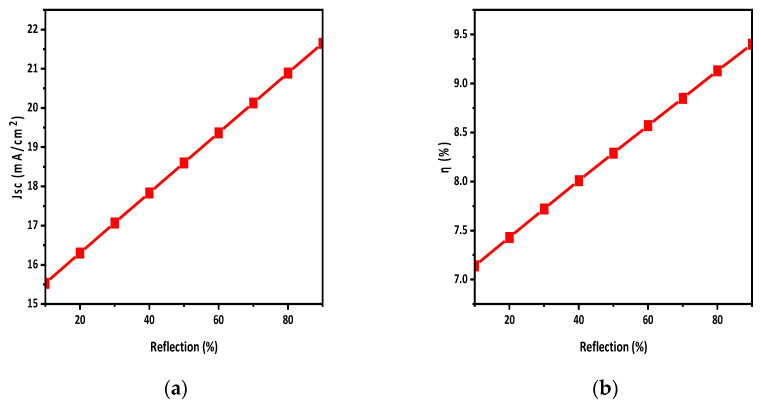
Effect of reflective coating on (**a**) Jsc; (**b**) η.

Back-side reflective coating is a key method for increasing the effectiveness of absorption capacity and enhancing overall efficiency. In this technique, a reflective coating is deposited on the back to enhance the optical path length. As we know, photons of larger wavelength (smaller energy) are absorbed by the active layer; however, sometimes they may not quite be absorbed. By adding the reflective coating, those photons are reflected and moved towards the absorber to increase the possibility of absorbance of those photons, which improves solar cell performance and efficiency [42]. In this simulation study, an aluminum metal coating was considered due to its high reflectivity in the visible and near-infrared region; however, gold (Au), and silver metal (Ag) can also be used. The back-side reflective coating was altered from 0 to 90% to study its effects. Figure 7a,b show that adding a reflective coating on the back side of the solar cell improves the Jsc from 14.763 mA/cm^2^ to 21.654 mA/cm^2^; as a result, the efficiency of the solar cell increases from 6.84 to 9.40.

### 3.6. Hole Transport Layer Doping Density

**Figure 8:**
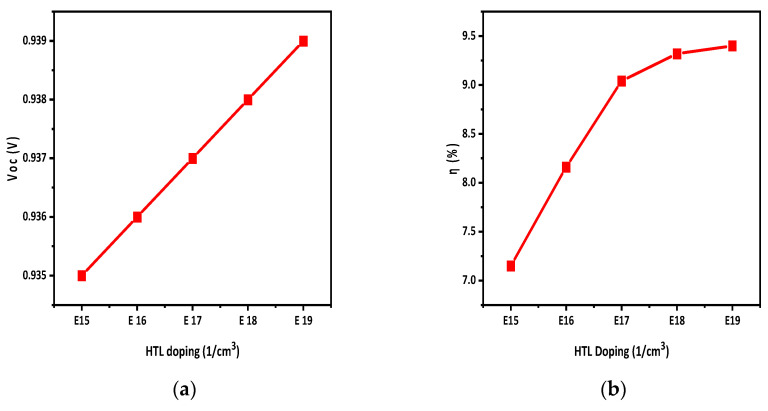
HTL doping density effect on (**a**) Voc; (**b**) η.

The main purpose of the CTL in organic solar cells is the facilitation of smooth ion movement from the active layer to the respective electrodes. For efficient transport, the CTL layer has maximum conductivity and minimum resistivity. This condition can be achieved through suitable doping. By increasing doping concentrations, the conductivity of the CTL improves, which enhances the electric field at the absorbing interface. As a result, the charge separation increases, which improves the efficiency (η) of solar cells. Some materials show a decline in performance at high doping due to the Moss–Burstein effect [33]. The HTL doping value was taken from the literature i.e., 2.8 × 10^19^ cm^−3^ [35]. So, the value of N_A_ was changed from 10^15^ to 10^19^ to examine this effect. Acceptor doping concentration affects the properties of solar cells, especially Voc and η. By increasing N_A_, the η and Voc of the structure was improved. The reason behind increasing the doping concentration was to boost the majority charge carriers, thereby enhancing the conductivity of the layer. This improved conductivity leads to a stronger electric field at the interface, promoting efficient ion separation. The impact of doping concentration on the Voc and η values is depicted in Figure 8a,b.

### 3.7. Electron Transport Layer Doping Effect

**Figure 9:**
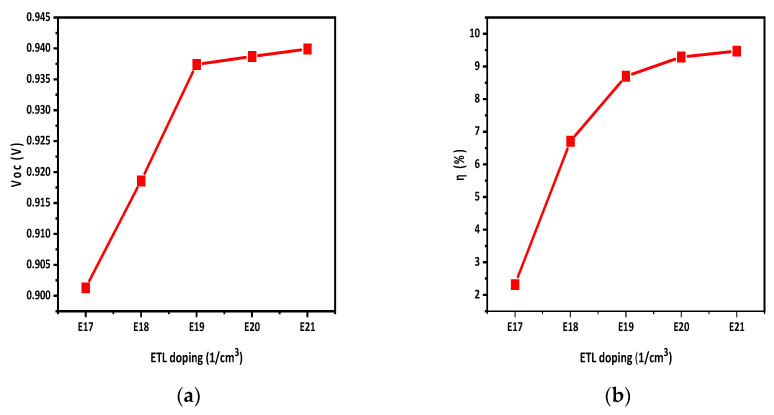
ETL doping density effect on (**a**) Voc; (**b**) η.

The ETL doping value was changed from E17 to E21 to observe the effect of doping concentration on the ETL. When the N_D_ value was changed from E17 to E21, the Voc and η increased, which is evident in Figure 9a,b. It is due to the majority charge carrier increases in the ETL which improve the conductivity and electric field of the layer.

### 3.8. Electric Field at Interface

**Figure 10:**
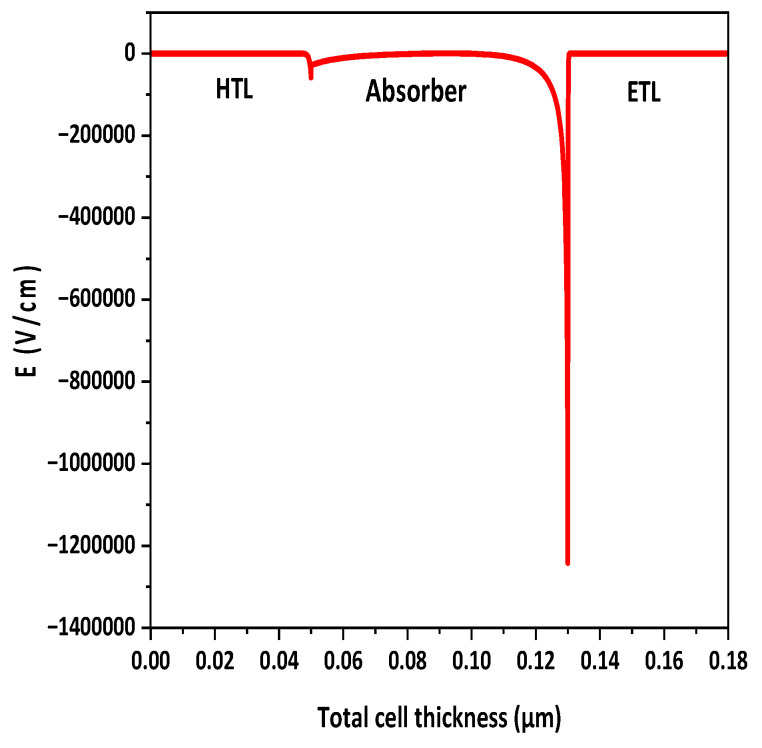
Distribution of electric potential at interfaces.

The efficiency of solar cells is significantly influenced by the electric field at the interface. When a high potential exists at the interface, charge carriers experience smooth separation and move toward their respective electrodes. Figure 10 illustrates the formation of two interfaces: the HTL/active layer and the ETL/active layer, each generating an electric field. The electric field at the ETL/active-layer interface is stronger than the HTL/active-layer interface; this may be due to high doping of the ETL. This discrepancy means that the ETL contributes to enhanced charge separation within the active layer.

### 3.9. Comparison of Simulation Results with Experimental Studies

These simulation results were compared with the experimental studies reported in [18] for the same active layer used in a photovoltaic device (ITO/PEDOT:PSS/PBDB-T:ITIC-OE/PFN-Br/Ag). It turned out that the obtained simulation results were in close agreement with the practical results. So, this software can be used for the performance enhancement of organic solar cells. The experimental and simulated results are shown in Table 6.

## 4. Conclusions

In this work, a BHJ polymer thin-film solar cell (FTO/PDINO/PBDB-T:ITIC-OE/spiro OMeTAD/Ni) was optimized and its efficiency enhanced by changing the thickness of the HTL and the active layer, doping level, and density of defects, through the presence of a back-side reflective coating. From these results, it was shown that by increasing the thickness of the active layer, Voc and FF decreased while Jsc and η increased. The reduction in Voc and FF is due to the increase in the series resistance with thickness, while Jsc and η increased due to the exciton formation. The active-layer thickness was optimized up to 80 nm. From the band gap-alignment diagram, it was concluded that the HTL had maximum CBO but minimum VBO, while the ETL had minimum CBO and maximum VBO, so there would be a smooth flow of electrons and holes.

The active-layer defect density has a noticeable impact on solar cell performance. By increasing the defect density, the Jsc of the solar cell decreases, which decreases the η of the solar cells. The effect of temperature on solar cells was also studied. By increasing the temperature, the performance of solar cells reduced, as η and Jsc decreased with the increase in temperature. A back-side reflective coating was applied to the back side of the solar cell which improved the efficiency and Jsc of the solar cell. The efficiency was increased by 2.5% by applying the back-side reflective layer, while the overall efficiency achieved was 9.4%. Moreover, it turns out that HTL and ETL doping improves the Voc and η of the solar cell. During the simulation, the HTL and ETL doping density varied from E15 to E19 and E17 to E21, respectively. It was observed that HTL doping increased V_oc_ from 0.935 to 0.939 and η from 7.15 to 9.4. Similarly, ETL doping raised Voc from 0.921 to 0.939 and η from 2.3 to 9.4.

Additionally, these obtained simulation results were compared with the practical results which are available in the literature. From these simulation studies, it was concluded that this software can be successfully used for the optimization and performance enhancement of organic solar cells. The results of our simulations confirm the roles of the presence of a reflective coating, thickness of the active layer, temperature, and the doping level on the photovoltaic parameters of (PBDB-T:ITIC-OE)-based BHJ OSCs.

## Figures and Tables

**Table 1:** Simulation parameters for active layer, HTL and ETL.

Parameters	Active Layer [18,34]	HTL [35]	ETL [36]
Thickness, d (nm)	80	50	50
Electron affinity, χ (eV)	4.03	2.2	4.110
Energy band gap, E_g_ (eV)	1.2	2.9	2.98
Dielectric permittivity, ε	6.1	3	5
Valence band effective density of states, N_V_ (cm^−3^)	1 × 10^19^	1 × 10^19^	1 × 10^19^
Conduction band effective density of states, N_C_ (cm^−3^)	1 × 10^19^	1 × 10^19^	1 × 10^19^
Electron thermal velocity, Vth_e_ (cm/s),	1 × 10^7^	1 × 10^7^	1 × 10^7^
Hole thermal velocity, Vth_p_ (cm/s),	1 × 10^7^	1 × 10^7^	1 × 10^7^
Electron mobility, μ_n_ (cm^2^/Vs)	1.2 × 10^−5^	1 × 10^−4^	2 × 10^−6^
Hole mobility, μ_p_ (cm^2^/Vs)	3.5 × 10^−4^	2 × 10^−4^	1 × 10^−3^
Donor density, N_D_ (1/cm^3^)	-	-	2 × 10^21^
Acceptor density, N_A_ (1/cm^3^)	-	2.8 × 10^19^	-
Defect density, N_t_ (1/cm^3^)	1 × 10^14^	1 × 10^14^	1 × 10^14^

**Table 2:** Back contact properties of electrode (anode).

Parameters	Values [26]
Thermionic emission velocity for electron	1 × 10^5^ cm/s
Thermionic emission velocity for hole	1 × 10^7^ cm/s
Back electrode work function, Ni	5.01 eV

**Table 3:** Front contact properties of electrode (cathode).

Parameters	Values [26,32]
Thermionic emission velocity for electron	1 × 10^7^ cm/s
Thermionic emission velocity for hole	1 × 10^5^ cm/s
Front electrode work function, (FTO)	4.4 eV

**Table 4:** Device performance parameters of optimized OSC before and after reflective coating.

Structure	Voc (V)	Jsc (mA/cm^2^)	FF (%)	η (%)
Before	0.927	14.763	49.98	6.84
After	0.939	21.654	46.2	9.40

**Table 5:** Obtained CBO and VBO of HTL and ETL values.

CTL	CBO	VBO
Spiro OMeTAD (HTL)	1.83	−0.13
PDINO (ETL)	−0.08	1.86

**Table 6:** Photovoltaic parameters determined for real structure and by simulation.

Parameters	Experimental [18]	Simulated
Voc	0.85	0.939
Jsc	14.8	21.654
FF	67.0	46.2
PCE	8.5	9.40

## Data Availability

Not applicable.
